# Zearalenone-Induced Interaction between PXR and Sp1 Increases Binding of Sp1 to a Promoter Site of the eNOS, Decreasing Its Transcription and NO Production in BAECs

**DOI:** 10.3390/toxins12060421

**Published:** 2020-06-25

**Authors:** Hyeon-Ju Lee, Jung-Hyun Park, Se-Young Oh, Du-Hyong Cho, Suji Kim, Inho Jo

**Affiliations:** 1 Department of Molecular Medicine, College of Medicine, Ewha Womans University, Seoul 07804, Korea; eihj0323@hanmail.net (H.-J.L.); piaflover@hanmail.net (J.-H.P.); ohs@ewha.ac.kr (S.-Y.O.); 2 Department of Pharmacology, College of Medicine, Yeungnam University, 170 Hyunchung-ro, Nam-gu, Daegu 42415, Korea; biohyong@hanmail.net (D.-H.C.); rlatnwl@yu.ac.kr (S.K.)

**Keywords:** zearalenone, eNOS, PXR, Sp1, NO

## Abstract

Zearalenone (ZEN) is a non-steroidal mycotoxin that has various toxicological impacts on mammalian health. Here, we found that ZEN significantly affected the production of nitric oxide (NO) and the expression of endothelial NO synthase (eNOS) of bovine aortic endothelial cells (BAECs). A promoter analysis using 5′-serially deleted human eNOS promoter revealed that the proximal region (−135 to +22) was responsible for ZEN-mediated reduction of the human eNOS promoter activity. This effect was reversed by mutation of two specificity protein 1 (Sp1) binding elements in the human eNOS promoter. A chromatin immunoprecipitation assay revealed that ZEN increased Sp1 binding to the bovine eNOS promoter region (−113 to −12), which is homologous to −135 to +22 of the human eNOS promoter region. We also found that ZEN promoted the binding of the pregnane X receptor (PXR) to Sp1 of the bovine eNOS, consequently decreasing eNOS expression. This reduction of eNOS could have contributed to the decreased acetylcholine-induced vessel relaxation upon ZEN treatment in our ex vivo study using mouse aortas. In conclusion, our data demonstrate that ZEN decreases eNOS expression by enhancing the binding of PXR-Sp1 to the eNOS promoter, thereby decreasing NO production and potentially causing vessel dysfunction.

## 1. Introduction

Zearalenone (ZEN) is an estrogenic mycotoxin from *Fusarium* fungi, and it is a common contaminant of cereal grains such as oat, barley, and sorghum worldwide. Due to the estrogenic activity of ZEN and its metabolites, some countries including the USA, Japan, and France have a guideline to limit the intake of ZEN, varying from 50 to 1000 μg/kg per day depending on the country [1]. Despite the regulation limit of ZEN for human food intake, humans are at risk of ZEN and its metabolites by intaking animal products. Mauro et al. reported that ZEN and its metabolites are present in the serum samples of nearly all surveyed participants, which were healthy women at ages 25–69 years recruited in Rutgers-New Brunswick, New Jersey, USA [2]. Their concentrations are positively correlated with meat intake and body mass index, suggesting that humans can easily be exposed to ZEN through the consumption of livestock fed with contaminated corn/grains, which could commonly occur in animal industries in any attempt to reduce production cost.

Many studies have pointed out the danger of ingesting ZEN and its metabolites, causing various toxic effects on reproductive cells/tissues [3,4,5,6] as well as hepatocytes and kidney cells [7,8]. Most toxicity studies of ZEN have been focused on animals, yet some reported toxic effects on human embryonic stem cells and SNO human esophageal carcinoma cells [9,10] due to the potential risk of ZEN exposure from intaking animal products [2]. Once ZEN is ingested by animals or humans, it must initially pass through hepatic and systemic blood vessels before reaching other target sites. Nevertheless, no such study has been published concerning vascular cells/tissues. To understand better the potential risk posed by ZEN exposure, it would be important to assess its potential toxicological effects on blood vessels as well as endothelial cells (ECs).

Endothelial cells (ECs) are an essential component of the cardiovascular system that constructs selective blood–tissue barriers of vascular tissues. ECs regulate vessel integrity by releasing its specific bioactive molecules, particularly nitric oxide (NO). NO is catalyzed by NO synthase (NOS), majorly endothelial NOS (eNOS) in ECs, and thus eNOS regulation is the key to maintain blood vessel homeostasis. Therefore, studying the regulation of eNOS by ECs is an important aspect to understand better about blood vessel function. The eNOS activity is commonly regulated by phosphorylation and dephosphorylation at its specific sites; for example, the phosphorylation of eNOS at serine 1179 (eNOS-Ser^1179^; in bovine sequence) increases eNOS activity and NO production, leading to vessel dilation [11,12,13,14]. On the contrary, the phosphorylation of either eNOS-Thr^497^ or eNOS-Ser^116^ decreases its activity leading to vessel constriction [15,16,17]. Apart from eNOS phosphorylation and dephosphorylation, eNOS activity can be regulated through changes in gene and protein expressions of eNOS itself, where 17β-estradiol increases eNOS protein and activity in ovine fetal pulmonary artery ECs [18].

Epidemiological studies showed that the prevalence of cardiovascular diseases dramatically increases in women after menopause, indicating a potential association of estrogen on vascular function. Considering that ZEN has estrogenic effects and ECs are likely to be exposed to ZEN once it is ingested by humans and animals before reaching other target tissues of ZEN, it is important to find potential toxic effects and mechanisms of ZEN on EC function. In this study, we looked at the effects of ZEN on eNOS activity and NO production using BAECs. We also characterized the mechanism underlying ZEN-induced alteration in eNOS activity and expression and then observed changes of ZEN-stimulated vessel relaxation using an ex vivo aortic mouse model.

## 2. Results

### 2.1. Zearalenone Decreases Nitric Oxide (NO) Production, Which is Accompanied by Decreased Protein and mRNA Expressions of eNOS

**Figure 1:**
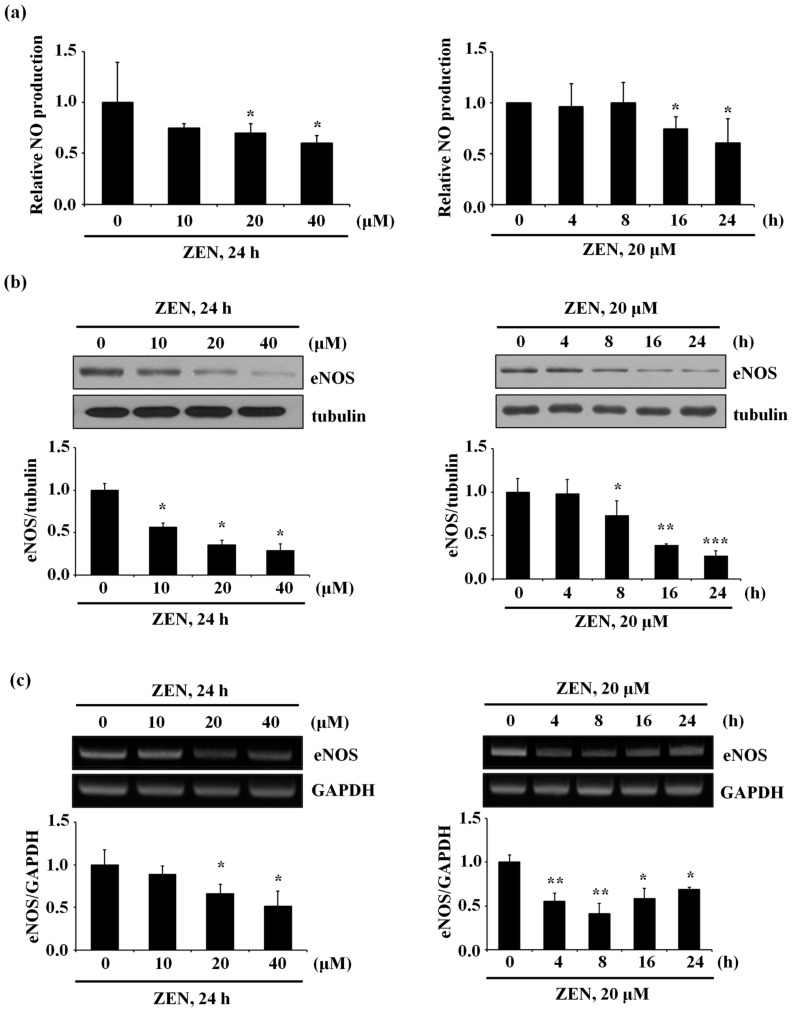
ZEN decreases NO production in a dose- or time-dependent manner, which is accompanied with the decreased protein and mRNA expressions of eNOS. BAECs were exposed to various concentrations of ZEN (0, 10, 20, 40 µM) for 24 h or 20 µM of ZEN for various time points (0, 4, 8, 16, 24 h). (**a**) NO production from the BAECs was measured by using the Griess method. (**b**) The protein expressions of eNOS relative to tubulin in the BAECs were quantified using Western blot analyses. (**c**) The mRNA expressions of eNOS relative to GAPDH in the BAECs were quantified using RT-PCR analyses. The plots depict the mean fold alterations below the controls (± S.D.) from at least four independent trials. Statistical significances are denoted as * *p* < 0.05, ** *p* < 0.01, and *** *p* < 0.001.

We found that ZEN significantly decreased both NO production (Figure 1a) and eNOS protein expression (Figure 1b) in a dose- and time-dependent manner in BAECs. Exposure with 20 μM of ZEN for 24 h reduced NO production to ~60%, while eNOS protein expression was reduced to ~30% compared with the control. ZEN also decreased mRNA expression of eNOS in a dose- and time-dependent manner (Figure 1c), where 20 µM of ZEN exposure for 8 h decreased eNOS mRNA expression to ~40% compared with the control. These data suggest that the decreased NO production and eNOS protein expression by ZEN might be attributed at least by the decreased eNOS mRNA expression. Since significant decreases in mRNA and protein expressions of eNOS were found in the BAECs exposed to 20 µM of ZEN for 8 and 24 h, respectively, all subsequent experiments were carried out under these conditions, unless mentioned otherwise.

### 2.2. Estrogen Receptors (ERs) Are Not Involved in the ZEN-Mediated Decrease in eNOS Protein Expression

**Figure 2:**
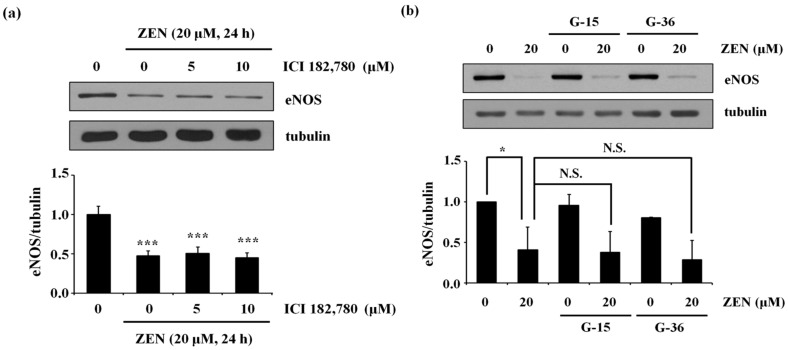
Estrogen receptor is not responsible for decreasing eNOS protein expression by ZEN. After pretreatment with various concentrations of (**a**) a genomic ER antagonist ICI 182,780 (0, 5, 10 µM), (**b**) a nongenomic ER antagonist G-15 (1 µM) or G-36 (1 µM) for 1 h, BAECs were exposed to 20 µM of ZEN for 24 h. The eNOS protein expression relative to tubulin was quantified using Western blot analyses. The plots depict the mean fold alterations below the controls (± S.D.) from at least four independent trials. Statistical significances are denoted as * *p* < 0.05, *** *p* < 0.001, and N.S. (not statistically significant).

Because ZEN has been reported to exhibit estrogenic-like effects via either genomic or nongenomic ER-mediated signaling pathways [3,19,20], we examined whether ERs in BAECs were also involved in the ZEN-mediated reduction of eNOS protein expression. As shown in Figure 2, neither ICI 182,780, a specific genomic ER antagonist, nor G-15 and G-36, specific nongenomic ER antagonists, affected the inhibitory effect of ZEN on eNOS protein expression. These data indicate that ERs are not involved in the ZEN-mediated decrease in eNOS protein expression.

### 2.3. The Proximal Promoter Region (−135 to +22) of the Human eNOS Gene is Involved in the ZEN-Mediated Decrease in eNOS Gene Transcription

**Figure 3:**
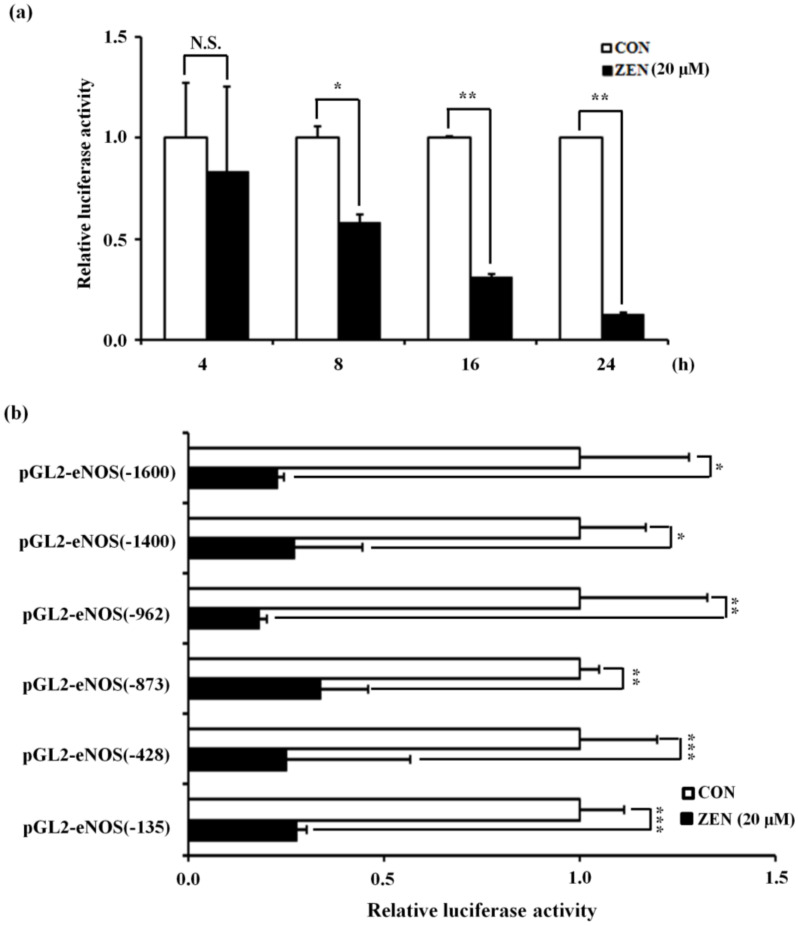
The proximal eNOS promoter (–135 to +22) is involved in ZEN-mediated decrease in eNOS transcriptional activation. (**a**) Luciferase activities of eNOS(−1600) vector and CMV promoter (renilla) in the BAECs exposed to 20 µM of ZEN for 4, 8, 16, 24 h. (**b**) Luciferase activities of the serially 5′-deleted promoters of human eNOS and CMV promoter (renilla) in the BAECs exposed to 20 µM of ZEN for 16 h. The plots depict the mean fold alterations below the controls (± S.D.). Statistical significances are denoted as * *p* < 0.05, ** *p* < 0.01, *** *p* < 0.001, and N.S. (not statistically significant).

Since our earlier experiments revealed that ZEN inhibited eNOS protein expression partially due to a decrease in eNOS mRNA expression, we examined whether ZEN decreased eNOS transcriptional activity. To this end, we measured eNOS promoter activity using the full-length human eNOS promoter region (−1600 to +22) and found that treatment with 20 µM ZEN for 8 h significantly reduced eNOS promoter activity (Figure 3a). To identify specific target site(s) for ZEN, we mapped region(s) of the human eNOS promoter gene responsible for the ZEN-mediated reduction in eNOS mRNA transcription. As shown in Figure 3b, progressive 5′-deletion from −1600, −1400, −962, −873, −428, to −135 did not reverse the ZEN-induced reduction of eNOS promoter activity, suggesting that the target site of ZEN is likely located in the proximal region in between −135 to +22 of the human eNOS promoter.

### 2.4. ZEN Decreases the Transcriptional Activation of eNOS by Increasing Sp1 Binding to −135 to +22 of the Human eNOS Promoter Region

**Figure 4:**
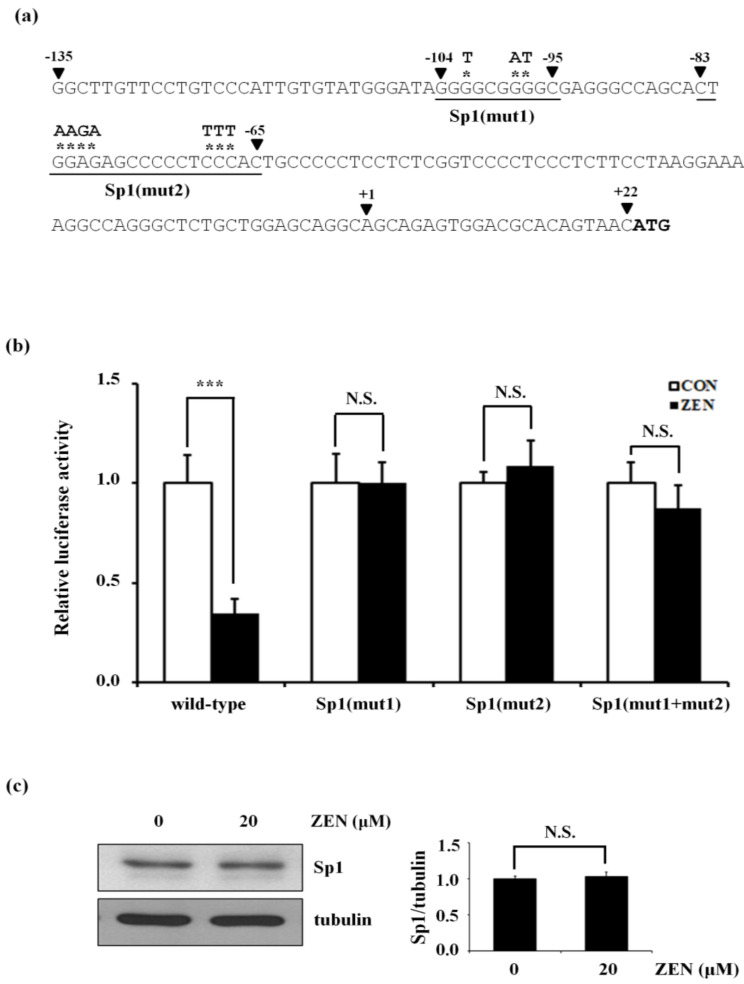
The Sp1 binding regions of the human eNOS promoter are related to the decreased eNOS promoter activity by ZEN. (**a**) The positions of the amino acid mutated, Sp1(mut1) and Sp1(mut2), are indicated with a black arrowhead, and the mutated amino acid is shown as a bolded uppercase with an asterisk (*). The method for site mutagenesis was described in Materials and Methods. (**b**) Luciferase activities of eNOS(−135) wild type or the mutants and CMV promoter (renilla) in the BAECs exposed to 20 µM of ZEN for 8 h. (**c**) The Sp1 protein expression relative to tubulin was quantified using Western blot analyses. The plots depict the mean fold alterations below the controls (± S.D.) from at least four independent trials. Statistical significances are denoted as *** *p* < 0.001 and N.S. (not statistically significant).

A previous study showed that Sp1 regulates eNOS expression by binding to the eNOS promoter G/C box region (−109 to −95 in the human eNOS promoter) [21]. From this study, we hypothesized that ZEN increases the binding of Sp1 to its target DNA site, i.e., G/C-rich regions within the human eNOS promoter (−135 to +22), consequently decreasing the mRNA transcriptional activity of eNOS. To validate this hypothesis, we first identified two potential Sp1 binding sites, −104 to −95 and −83 to −65, within the human eNOS promoter (−135 to +22) using the online tool AliBaba2.1 [22] (Figure 4a). To test whether these sites are associated with the effects of ZEN on decreased human eNOS promoter activity, we mutated several nucleotides within these identified sites (indicated by the bolded uppercase letters and asterisks in Figure 4a). These mutations recovered the ZEN-induced decrease in eNOS promoter activity to levels close to those of the control group not exposed to ZEN (Figure 4b). We also found that ZEN did not increase Sp1 protein expression under our experimental conditions (Figure 4c). All these data suggest that ZEN does not alter Sp1 expression but rather induces binding of Sp1 to the human eNOS promoter at either −104 to −95, −83 to −65, or both regions, thereby decreasing eNOS promoter activity. These results indicate that Sp1 functions as a repressor of eNOS promoter activity under our conditions.

### 2.5. Mithramycin A, a Selective Sp1 Inhibitor, and Sp1 siRNA Can Reverse the ZEN-Mediated Decrease in eNOS Promoter Activity, Protein Expression, and NO Production

**Figure 5:**
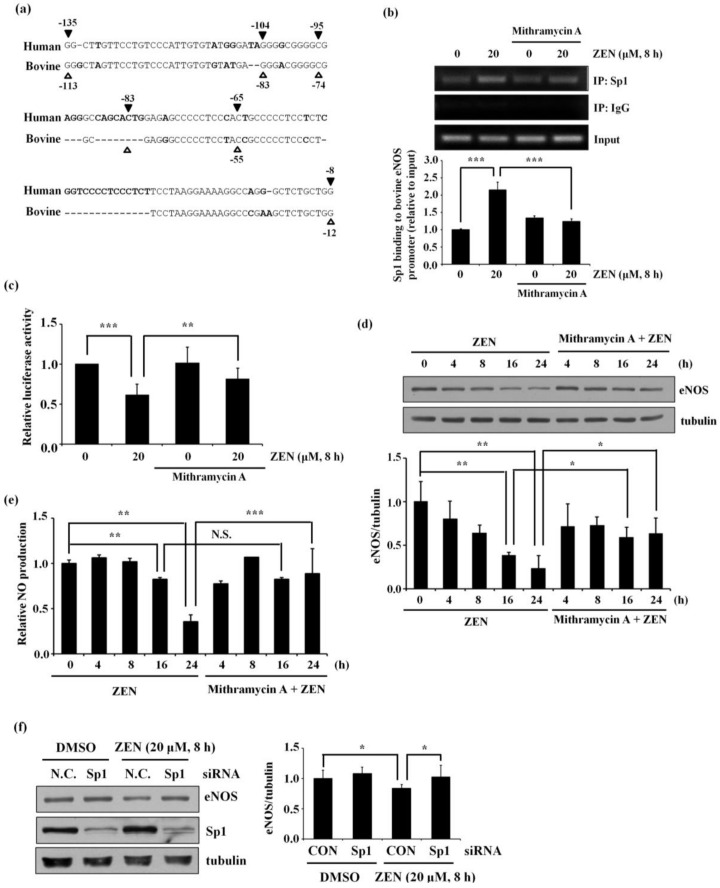
ZEN increases the binding of Sp1 to the bovine eNOS promoter region (−113 to −12), thereby decreasing the promoter activity and protein expression of eNOS. (**a**) Human and bovine eNOS promoters were aligned using the Multalin online tool (http://multalin.toulouse.inra.fr/multalin/). The unmatched amino acid residues within the homologous regions between human (▼) and bovine (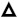) are indicated in bold. (**b**) After pretreatment with vehicle or 20 nM of the Sp1 inhibitor mithramycin A for 1 h, BAECs were further co-treated with or without 20 μM of ZEN for 8 h. Binding of Sp1 to the bovine eNOS promoter gene relative to the total chromatin extract (Input) was quantified using a ChIP assay, as described in Materials and Methods. (**c**) Luciferase activities of the bovine *eNOS*(−135) vector and CMV promoter (*Renilla*) in BAECs treated with vehicle or 20 nM mithramycin A for 1 h, followed by further co-treatment with 20 µM of ZEN for 8 h. (**d**) Protein expression of eNOS relative to tubulin was quantified using Western blot analyses, and (**e**) NO production was quantified using the Griess method in BAECs treated with vehicle or 20 nM mithramycin A followed by further co-treatment with 20 µM ZEN for 4, 8, 16, and 24 h. (**f**) Protein expression of eNOS relative to tubulin was quantified using Western blot analyses of BAECs transfected with siRNA targeting Sp1 for 24 h and then exposed to 20 µM of ZEN for 8 h. N.C., negative control. Plots depict mean fold alterations from the control (± S.D.) from at least four independent trials. Statistical significance is denoted as follows: * *p* < 0.05, ** *p* < 0.01, *** *p* < 0.001, and N.S. (not statistically significant).

To further explore how ZEN decreases eNOS promoter activity via Sp1, we used mithramycin A and siRNA to selectively inhibit Sp1 binding to G/C-rich DNA sequences of the target gene [23]. To this end, we first used the Multalin online tool (http://multalin.toulouse.inra.fr/multalin/) to align human and bovine eNOS promoter sequences, and we found that −113 to −12 of the bovine eNOS promoter corresponded to −135 to −8 of the human eNOS promoter (Figure 5a). We then designed primers using the bovine eNOS promoter sequence. As shown in Figure 5b, ChIP assay revealed that treatment with mithramycin A significantly reversed ZEN-stimulated Sp1 binding to the bovine eNOS promoter. As expected, mithramycin A also reversed the ZEN-mediated decrease in eNOS promoter activity (Figure 5c), eNOS protein expression (Figure 5d), and NO production (Figure 5e). Lastly, when Sp1 was knocked down using specific siRNA, the ZEN-mediated decrease in eNOS protein expression was significantly reversed (Figure 5f). Together, these results indicate that ZEN-stimulated Sp1 binding to specific G/C-rich sequences (−113 to −12) of the bovine eNOS promoter could reduce NO production as well as the expression of eNOS protein in BAECs.

### 2.6. Histone Deacetylases (HDACs), NCoR1, or SMRT Do Not Reverse the ZEN-Stimulated Decrease in the Expression of eNOS Protein and mRNA

**Figure 6:**
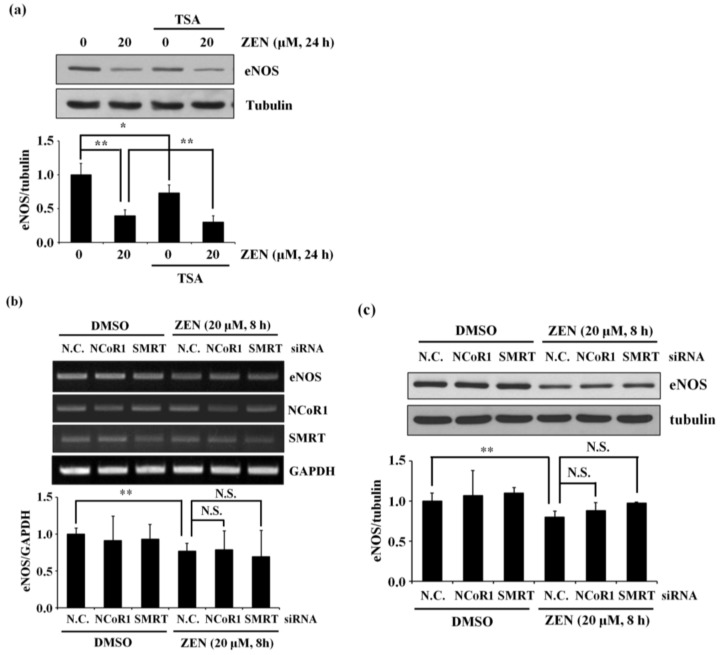
HDACs, NCoR1, and SMRT do not affect the ZEN-mediated decrease in mRNA and protein expression of eNOS. (**a**) The protein expression of eNOS relative to tubulin was quantified using Western blot analyses in the BAECs exposed to 20 µM of ZEN for 24 h after pretreatment with vehicle or HDAC inhibitor TSA for 1 h. The plots are representative of four independent experimental trials. (**b**) The mRNA expression of eNOS relative to GAPDH or (**c**) the protein expression of eNOS relative to tubulin was quantified using RT-PCR and Western blot analyses in the BAECs transfected with siRNA of NCoR1 or SMRT for 24 h, respectively, 20 µM of ZEN exposure for 24 h. N.C., negative control. The plots depict the mean fold alterations below the controls (± S.D.) from four independent trials. Statistical significances are denoted as * *p* < 0.05, ** *p* < 0.01, and N.S. (not statistically significant).

Several studies have shown that HDACs function as cofactors to regulate Sp1 expression [24,25]. Therefore, we first explored whether HDACs are involved in the ZEN-mediated decrease in eNOS protein expression using TSA, an HDAC inhibitor. As shown in Figure 6a, TSA did not reverse the ZEN-mediated decrease in eNOS protein level. Treatment with TSA alone (without ZEN) also slightly reduced eNOS protein expression. Previous studies have also reported that Sp1 affects the expression of target genes via the nuclear receptor corepressors, NCoR1 and SMRT [26,27]. Therefore, we tested whether NCoR1 and SMRT are associated with the ZEN-mediated decrease in eNOS expression. When NCoR1 and SMRT were knocked down using their corresponding siRNAs, however, the ZEN-stimulated decrease in mRNA and protein expression of eNOS was not affected (Figure 6b,c). These data indicate that the nuclear corepressors as well as HDACs tested in this study are not associated with the ZEN-mediated decrease in eNOS expression.

### 2.7. Pregnane X Receptor (PXR) Induces the ZEN-Mediated Decrease in eNOS mRNA and Protein Expression

**Figure 7:**
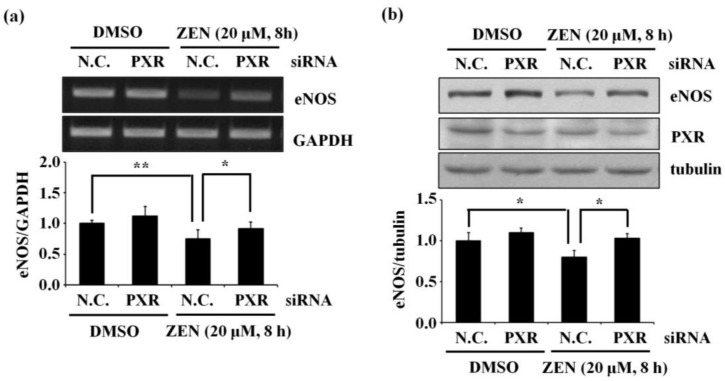
PXR is associated with the ZEN-mediated decrease in mRNA and protein expression of eNOS. (**a**) mRNA expression of eNOS relative to GAPDH and (**b**) protein expression of eNOS relative to tubulin were quantified using RT-PCR and Western blot analyses, respectively, in BAECs transfected with siRNA targeting PXR with or without 20 µM of ZEN exposure for 8 h. N.C., negative control. Plots depict mean fold alterations from the control (± S.D.) from at least four independent trials. Statistical significance is denoted as * *p* < 0.05 and ** *p* < 0.01.

We confirmed that inhibition of HDACs and knockdown of NCoR1 and SMRT did not reverse the effect of ZEN on the expression of eNOS (Figure 6). However, ZEN has been shown to regulate CYP3A4 expression through PXR [28], and changes in PXR are known to affect eNOS protein expression [29]. Based on these previous studies, we evaluated whether ZEN affects eNOS expression by altering PXR expression. As shown in Figure 7, when PXR was knocked down, the ZEN-induced decrease in levels of eNOS mRNA and protein was attenuated, indicating a role for PXR in the ZEN-mediated decrease in eNOS expression.

### 2.8. ZEN-Stimulated PXR Binding to Sp1 Is Required for Binding of Sp1 to the eNOS Promoter of BAECs

**Figure 8:**
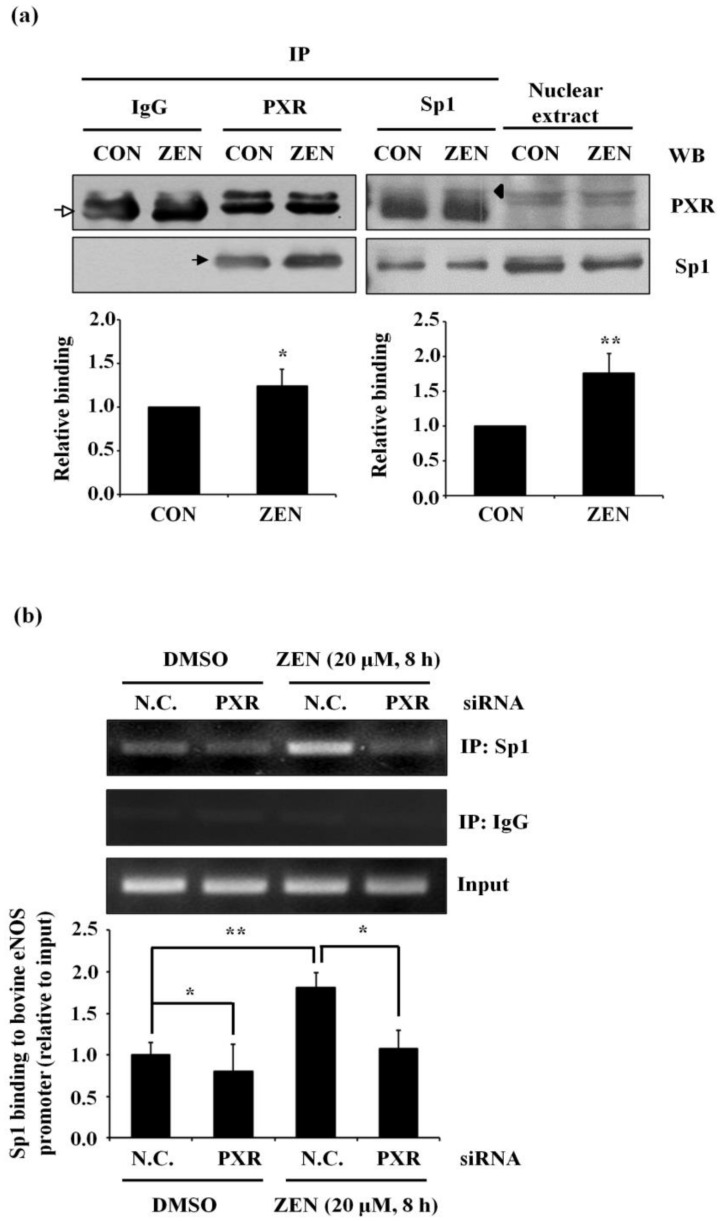
ZEN induces the binding of PXR to Sp1, increasing Sp1 binding to the bovine eNOS promoter region. (**a**) BAECs were exposed to 20 μM ZEN for 8 h, and nuclear fractions were extracted. Nuclear fractions were subjected to co-IP assays using antibodies against PXR, Sp1, or non-immune IgG. Binding of proteins was measured by Western blot analyses, and the blots shown are representative of four independent experimental trials. The arrowhead indicates PXR protein, while the filled arrow and the blank arrow indicate Sp1 protein and heavy chain, respectively. (**b**) After the transfection of BAECs with siRNA targeting PXR, cells were incubated with or without 20 μM ZEN for 8 h. Binding of Sp1 to the bovine eNOS promoter relative to the total chromatin extract (Input) was quantified using a ChIP assay as described in Materials and Methods. N.C., negative control. Plots depict mean fold alterations from the control (± S.D.) from at least four independent trials. Statistical significance is denoted as follows: * *p* < 0.05 and ** *p* < 0.01.

In our earlier experiments, we found that knockdown of PXR reversed the effect of ZEN on the expression of eNOS. To test whether this reversal is also mediated by Sp1, we first performed a co-IP assay. As shown in Figure 8a, PXR bound Sp1 in basal BAECs, and ZEN significantly increased the binding between PXR and Sp1. Furthermore, ChIP assay revealed that knockdown of PXR significantly decreased ZEN-mediated Sp1 binding to the bovine eNOS promoter (Figure 8b). Knockdown of PXR also decreased the level of Sp1 binding to the bovine eNOS promoter in basal BAECs. From these results, ZEN appears to induce binding of PXR to Sp1, which subsequently increases Sp1 binding to the bovine eNOS promoter, resulting in decreased eNOS expression in BAECs.

### 2.9. ZEN Decreases Acetylcholine (ACh)-Induced Vessel Relaxation Accompanied by Decreased eNOS Protein Expression in Mouse Aortas

**Figure 9:**
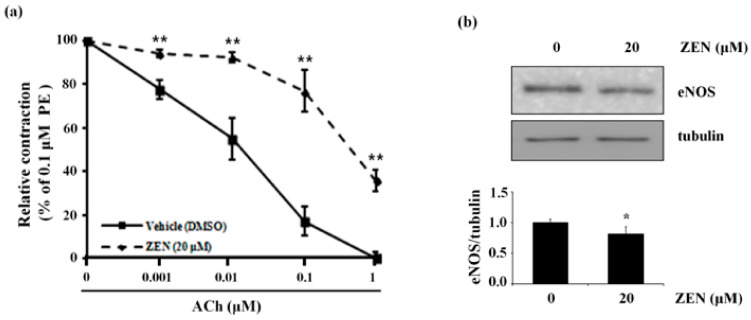
ZEN reduces endothelium-dependent vessel relaxation and eNOS protein expression in mouse aortas. Endothelium-intact aortas were dissected from male C57BL/6 mice at 8 weeks old. Mouse thoracic aortas were prepared, and a vessel relaxation assay was performed as described in Materials and Methods. (**a**) Vessel relaxation indexes of endothelium-intact aorta rings exposed to 20 μM of ZEN or vehicle for 16 h. Aorta rings were contracted with 0.1 μM phenylephrine (PE), followed by cumulative treatment with acetylcholine (ACh, 0.001–1 μM). The line plots represent the mean ± S.E. at each point (*n* = 6). (**b**) eNOS protein expression relative to tubulin was quantified by Western blot analyses of total protein from each aorta ring exposed to 20 µM of ZEN or vehicle for 16 h. Plots depict mean fold alterations from the control (± S.D.) from three independent trials. Statistical significance is denoted as follows: * *p* < 0.05 and ** *p* < 0.01.

To validate the results from the in vitro studies, we looked at the effect of ZEN on ACh-induced vessel relaxation using an isolated mouse aorta ex vivo model. Pretreatment of aortas with ZEN (20 μM for 16 h) significantly decreased ACh-induced aortic vessel relaxation compared with the DMSO-pretreated control (*n* = 6) (Figure 9a). Simultaneously, ZEN also significantly decreased eNOS protein expression in mouse aortas compared with the control (Figure 9b). Taken together, our results suggest that ZEN decreases aortic vessel relaxation stimulated by ACh, probably by downregulating eNOS expression of the vessel cells as we observed in our in vitro experiments.

## 3. Discussion

eNOS is a key regulator of NO production, which is involved in maintenance of blood vessel homeostasis [30,31]. Many studies have demonstrated that phosphorylation/dephosphorylation of eNOS at specific sites is a major regulatory mechanism to alter eNOS activity. Some studies have also reported that eNOS activity can be affected by changes in protein and mRNA expression of eNOS upon exposure to external stimuli. For example, H_2_O_2_ increases the stability of eNOS mRNA, thereby increasing eNOS mRNA transcription and activity [32]. On the contrary, tumor necrosis factor-α promotes binding of eukaryotic elongation factor 1-α 1 (eEF1A1) [33] or microRNA-155 [34] to the 3′-UTR of eNOS mRNA, thereby reducing mRNA stability and decreasing eNOS expression. Our current findings indicate that ZEN can decrease eNOS activity by different mechanisms from those reported previously. In particular, our results demonstrate that ZEN promotes binding of PXR to Sp1, which then increases the binding affinity of Sp1 to specific G/C-rich regions of the eNOS promoter (−113 to −12), consequently decreasing eNOS expression in BAECs.

The human eNOS promoter has positive regulatory domains I (PRD I; −104 to −95) and II (−144 to −115) where transcription factors such as Ets-1, YY1, Elf-1, MAZ, and Sp1 bind to regulate eNOS mRNA transcription [32]. The results from our study demonstrate that Sp1 could function as a repressor of eNOS mRNA transcription to decrease eNOS expression. In this regard, oxidative stress has also been reported to decrease eNOS expression by increasing Sp1 binding to specific human eNOS promoter regions (−1386, −632, −104) [35]. Our results were largely consistent with these previous study results with a few exceptions; oxidative stress has been reported to increase the level of Sp1 protein and its phosphorylation, yet we failed to find any alteration in Sp1 protein expression in BAECs treated with ZEN. These differences are probably because of the use of different cell types and external stressors. The authors in the previous study used human hepatocarcinoma cells transfected with the human eNOS promoter gene, while we used BAECs naturally expressing eNOS. Furthermore, it is difficult to compare directly the results of the two studies since different stressors were used. It would be interesting to investigate the reason for these differences further, but this is beyond the scope of the current study.

Understanding the detailed molecular mechanism by which Sp1 inhibits eNOS promoter activity warrants further investigation. Various cofactors expressed in response to external stimuli interact with Sp1 to regulate eNOS promoter activity; for example, HDACs, NCoR1, and/or SMRT regulate gene activity by binding to Sp1. Binding of HDAC1 to Sp1 decreases murine thymidine kinase (TK) promoter activity in Swiss 3T3 fibroblasts [25], while HDAC1 has been shown to compete with E2F1 for binding to the carboxyl-terminal of Sp1 in the regulation of murine TK promoter activity [24,25]. Other studies showed that NCoR1 and SMRT bind to Sp1 to regulate the promoter activities of the vascular endothelial growth factor receptor 2 (VEGFR2) gene in MCF-7 cells [27] and the UAS gene in African green monkey kidney cells (CV-1) [26], respectively. In contrast to these previous studies, none of these transcription factors including HDAC1, NCoR1, and SMRT affected the ZEN-mediated decrease in eNOS protein and mRNA in BAECs.

Instead, we found that ZEN induced an interaction between PXR, a ligand-activated nuclear receptor, and Sp1, thereby altering eNOS expression. PXR is primarily known as a sensor of exogenous chemicals that can regulate gene expression, enzyme biotransformation, and drug transportation [36,37,38]. Similar to these studies, ZEN mediates the expression of xenobiotic-inducible genes such as CYP3A through increasing PXR activation in human hephatocellular carcinoma HepG2 cells [28]. At the same time, our study clearly showed that PXR could function as a corepressor of eNOS transcription. In support of this, PXR was shown to suppress the transcription of the rifampicin-mediated G6Pase gene by directly interfering with the binding of cAMP response element-binding protein (CREB) to the CRE response element of this gene in Huh7 cells [39]. Another study also reported that PXR had a negative effect on eNOS expression; indole 3-propionic acid, a ligand of PXR, decreased eNOS expression in mice with an intact PXR but not mice with PXR knocked out, indicating the importance of PXR in regulating eNOS expression. Consistent with this recent study, we found firstly that PXR indirectly inhibits eNOS expression in ECs through interaction with Sp1.

So far, we have discussed how both Sp1 and PXR can decrease eNOS expression. The next question was whether Sp1 and PXR interact physically. When ECs were exposed to ZEN, we found that PXR interacted with Sp1 as a cofactor to decrease eNOS expression, indicating that it affects the gene transcription of eNOS indirectly by altering the binding affinity of Sp1 to the promoter of eNOS. Several studies have reported that cofactors of Sp1 bind to the Sp1 inhibitory domain (ID) or C2H2-type zinc finger DNA-binding domain (ZFDBD) to regulate Sp1 binding affinity, thereby changing target gene expression [26]. Additionally, some ligand-activated receptors such as the thyroid hormone receptor, retinoic acid receptor, and vitamin D_3_ receptor (VDR) retain a DNA binding domain (DBD) and ligand-binding domain (LBD). These domains are important for interaction with Sp1 to allow a complex of nuclear receptors to form; this complex subsequently binds to G/C-rich regions in the promoter of IL-1β, altering gene transcriptions [40]. In addition, VDR was shown to bind to C-terminal sites of Sp1 (622 to 788). Similar to these receptors, PXR also retains a DBD and LBD [41], suggesting that PXR is likely to interact with Sp1 to increase the affinity of Sp1 for G/C-rich regions of the eNOS promoter. Therefore, we speculate that ZEN promotes binding of PXR to Sp1, which increases the binding affinity of Sp1 for the bovine eNOS promoter region (−113 to −12), resulting in decreased transcription of eNOS in BAECs. The ZEN-medicated decrease in expression of eNOS protein and NO production would eventually decrease vessel relaxation, as we observed in our ex vivo mouse aortic model. Nonetheless, the concentration of ZEN used in this ex vivo study was higher compared to those circulating in animals exposed to ZEN-contaminated food [42]. Different cells and animals respond differently to a wide range of concentrations of ZEN, causing diverse effects. Like concentration ranges of ZEN between 10 pM and 300 μM used in many in vitro studies [43], the adequate concentrations responsible for in vivo toxicological effects are difficult to define due to a variety of times, doses, and routes of injections. A recent in vivo study showed that intratesticular injection of 300 ng ZEN/day (estimated to be 0.5 μM/day) for 21 days decreased regeneration of Leydig cells [44]. Considering that continuous exposure of ZEN could lead to accumulation in host tissues [45], there is a potential chance that chronic exposure of ZEN for a long time may lead to a similar effect as what we observed in our study, disrupting vascular function.

It is also interesting to note that the observed effects of ZEN on eNOS expression were independent of ERs in our study. ZEN has been reported to bind competitively to genomic or nongenomic ERs and then activate transcription of several estrogen-responsive genes [46,47,48]. However, we found that the effect of ZEN on eNOS expression was mediated by neither genomic nor nongenomic ERs in BAECs. Another study reported that ZEN induces neutrophil extracellular trap production via NADPH oxidase, extracellular signal-regulated kinase, and p38-dependent signaling pathways, but not through genomic ER signaling pathway in primary bovine neutrophils [49]. Although the previous study did not examine the involvement of nongenomic ER, our data suggest that some toxicological effects of ZEN, including the reduction in eNOS expression, are likely to be mediated via other receptors or molecules that are independent of ER-mediated signaling pathways. We did not identify specific upstream target(s) of binding of PXR to Sp1 in ZEN-inhibited eNOS expression in the current study; these targets should be identified in future studies.

## 4. Conclusions

**Figure 10:**
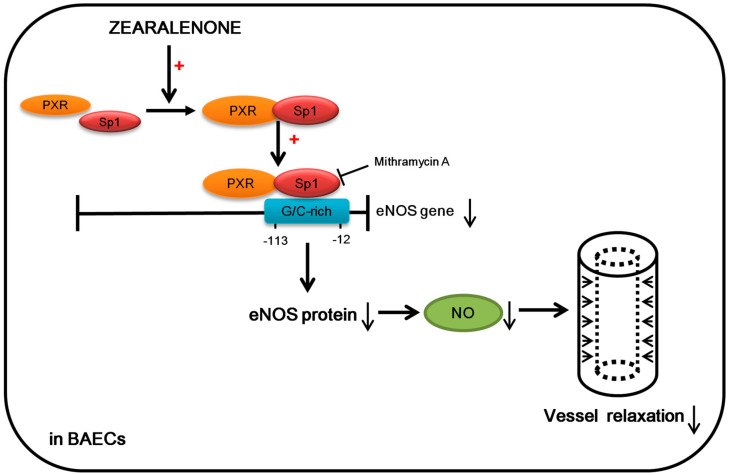
A schematic illustration of the molecular mechanism underlying the ZEN-mediated decrease in eNOS gene expression and NO production in BAECs and mouse aortas. ZEN increases the interaction between PXR and Sp1, which decreases eNOS gene transcription through increasing binding of the PXR-Sp1 complex to a G/C-rich region (−113 to −12) in the bovine eNOS promoter. This effect decreases eNOS protein expression and NO production in BAECs, and vessel relaxation in mouse aortas.

In this study, we found that ZEN significantly inhibited eNOS expression and NO production in BAECs, partially by decreasing eNOS gene transcription. The eNOS transcription is downregulated as the ZEN induced the affinity interaction between PXR and Sp1, consequently increasing binding of the Sp1 to the proximal region (−113 to −12) of the bovine eNOS promoter. This seems to be one of major molecular modes of ZEN to mediate the toxicological effects in decreasing the vessel relaxation of mouse aortas as illustrated in Figure 10. Therefore, these findings suggest that ZEN impairs the NO-related vascular system by inducing the affinity of PXR-Sp1 complex to eNOS promoter site, presenting a potential novel risk of ZEN exposure in relation to vascular health.

## 5. Materials and Methods

### 5.1. Materials

Zearalenone (ZEN), trichostatin A (TSA, HDAC inhibitor), and mithramycin A (Sp1 inhibitor) were purchased from Merck Millipore (Darmstadt, Germany). ICI 182,780 (genomic estrogen receptor (ER) antagonist) and G-15 (nongenomic ER antagonist) were acquired from Tocris bioscience (Bristol, UK). G-36 (nongenomic ER antagonist) was acquired from Cayman Chemical (Ann Arbor, MI, USA). eNOS-specific antibody was acquired from BD Transduction Laboratories (Lexington, KY, USA). α-tubulin antibody was acquired from AbFrontier (Seoul, Korea). Antibodies for Sp1 and pregnane X receptor (PXR) as well as all HRP-conjugated secondary antibodies were obtained from Santa Cruz Biotech (Santa Cruz, CA). Minimal essential medium (MEM), newborn calf serum (NCS), Dulbecco’s phosphate-buffered saline (DPBS), L-glutamine, penicillin–streptomycin antibiotics, and trypsin-EDTA were acquired from Thermo Fisher Scientific (Waltham, MA, USA).

### 5.2. Full-Length and 5′-Deleted Human eNOS Promoter

Full-length human eNOS promoter (−1600 to +22; *eNOS*(−1600)) and serially 5′-deleted promoters of *eNOS*(−1400), *eNOS*(−962), *eNOS*(−873), and *eNOS*(−428) were prepared as described previously [50]. *eNOS*(−135) was amplified by polymerase chain reaction (PCR) with the following forward (F) and reverse (R) primers: *eNOS*(−135)-F, 5′-GGC TTG TTC CTG TCC CAT TG-3′ and *eNOS*(−135)-R, 5′-TGC TGC CTG CTC GAG CAG AGC -3′. Synthesized PCR products containing each progressive 5′-deleted human eNOS promoter sequence were inserted into a luciferase reporter gene plasmid (pGL2). Corresponding *eNOS*–luciferase promoter constructs were designated pGL2-*eNOS*(−1600), pGL2-*eNOS*(−1400), pGL2-*eNOS*(−962), pGL2-*eNOS*(−873), pGL2-*eNOS*(−428), and pGL2-*eNOS*(−135), respectively.

### 5.3. Site-Directed Mutagenesis

Site-directed mutagenesis of pGL2-*eNOS*(−135) plasmid was prepared using a QuikChange II site-Directed Mutagenesis kit (Agilent Technologies, Santa Clara, CA, USA) according to the manufacturer’s protocol. Sequences of mutagenic primer pairs were as follows (mutagenized bases are identified by lowercase letters): *Sp1*(mut1)-F, 5′-GTG TAT GGG ATA GGt GCG atG CGA GGG CCA GCA C-3′; *Sp1*(mut1)-R, 5′-GTG CTG GCC CTC GCa TCG caC CTA TCC CAT ACA C-3′; *Sp1*(mut2)-F, 5′-CGG GGC GAG GGC CAG CAC Taa gaA GCC CCC Ttt tAC TGC CCC CTC- 3′; *Sp1*(mut2)-R, 5′-ACC GAG AGG AGG Gct CAG TGt cAG GGG cCT CTC CAG TGC TG-3′.

### 5.4. Cell Culture and Drug Treatments

BAECs were obtained as previously described [51] and cultured in MEM supplemented with 5% NCS in a humidified atmosphere chamber containing 5% CO_2_ at 37 °C. BAECs established a typical cobblestone structure of ECs and EC-specific marker von Willebrand factor VIII. BAECs between passages 5 and 9 were used in all experimental trials. When the cells were at ~80% confluence, 1% NCS MEM containing the indicated concentrations of ZEN was added. In some experimental trials, the cells were pretreated various factors for 1 h prior to ZEN exposure.

### 5.5. Transfection

BAECs were transfected with 3 μg of pGL2-*eNOS*(−1600), pGL2-*eNOS*(−1400), pGL2-*eNOS*(−962), pGL2-*eNOS*(−873), pGL2-*eNOS*(−428), or pGL2-*eNOS*(−135) using Lipofectamine 2000 (Thermo Fisher Scientific) according to the manufacturer’s instructions. For all pGL2 transfection experiments, BAECs were cotransfected with 70 ng of *Renilla* luciferase reporter vector for normalizing transfection efficiency. For small interfering RNA (siRNA) transfection, two or three potential siRNA oligonucleotides for nuclear receptor corepressor 1 (NCoR1), silencing mediator of retinoid and thyroid hormone receptor (SMRT; also referred to as NCoR2), and pregnane X receptor (PXR) were purchased from GenePharma (Shanghai, China) and mixed for better efficiency. siRNA oligonucleotide sequences were as follows: *NCoR1*, 5′-CAU UUG GAG UCA AAC AUG AAG-3′, 5′-GAA GAA AAA GUA GAA GAA AAG-3′, 5′-GUC AAU CUG CCA UCA AAC ACA-3′; *SMRT*, 5′-CCA UGA AGG UGU ACA AAG ACC-3′, 5′-GGG AAA AGA CUC AAA GUA AAC-3′; *PXR*, 5′-CUG GUU AUC ACU UCA AUG UCA UG-3′, 5′-GGG AAG AUC UGU GUU CAA UGA AG-3′, 5′-AUC AUU ACA CAC UGA CAA UAA GC-3′. Negative control siRNA oligonucleotide with the sequences 5′-UUC UCC GAA CGU GUC ACG UTT-3′ was also obtained from GenePharma. Commercially available siRNAs targeting Sp1, PXR, and negative control siRNA were obtained from Santa Cruz. siRNA (100 nM) was transfected to BAECs using DharmaFECT 4 transfection reagent (Horizon Discovery Ltd. Cambridge, UK) according to the manufacturer’s protocols.

### 5.6. Luciferase Assay

After the transfection of pGL2-*eNOS* plasmids, the transfected cells were maintained for 24 h, washed twice with DPBS, and lysed by adding 200 μL of 1 × passive lysis buffer (Promega, Madison, WI, USA). Lysates were collected in 1.5 mL tubes and centrifuged at 16,000× *g* for 20 min. Protein (20 μg) in a total of 20 μL supernatant was used for the luciferase assay. The Dual-Luciferase Reporter Assay System (Promega) was used to assess the luciferase activity of the transfectants. All data from firefly luciferase were normalized with *Renilla* luciferase.

### 5.7. Reverse Transcription (RT)-PCR

BAECs were homogenized with 1 mL of TRIzol reagent (Thermo Fisher Scientific) to extract total RNA from BAECs as previously described [12]. The total RNA was then converted to cDNA using SuperScript^TM^ III reverse transcriptase (Thermo Fisher Scientific). PCR for each target gene was carried out using the following primers: *eNOS*-F, 5′-GCC TCC TGT GAG ACC TTC TG- 3′; *eNOS*-R, 5′- TCT CTG GGA AGT CAC CTT GG- 3′; *NCoR1*-F, 5′- GAA ACA CCT GGC GAT GCT AT- 3′; *NCoR1*-R, 5′- GCT TCT CAT GCA CAA CAG GA- 3′; *SMRT*-F, 5′- TCT GGG GAA GAC AAT GAT GA-3′; *SMRT*-R, 5′- GTG TCG GAG CTG TTG TTG AC-3′; *PXR*-F, 5′- TAG GTC TGT GGA GAG CCA AG-3′; *PXR*-R, 5′- CCA GGA TCC ATG TCT GAG TC-3′; *GAPDH*-F, 5′- TCA CCA GGG CTG CTT TTA AT- 3′; *GAPDH*-R, 5′- GGT CAT AAG TCC CTC CAC GA- 3′. The amplified gene products were separated using a 1.0% agarose gel in TAE buffer (40 mM Tris-acetate, pH 8.0, 1 mM EDTA), tagged with RedSafe^TM^ Nucleic acid Staining Solution (iNtRON Biotechnology, Gyeonggi-do, Korea), and visually observed under UV.

### 5.8. Western Blot Analysis

BAECs exposed to ZEN with or without various pretreatments were washed with ice-cold DPBS. The cells were then lysed with lysis buffer (20 mM Tris-HCl at pH 7.5, 1% Triton X-100, 150 mM NaCl, 1 mM EDTA, 1 mM EGTA) supplemented with Protease Inhibitor Cocktail^TM^ (Merck Millipore), 1 mM NaF and 1 mM Na_3_VO_4_, 1 mM β-glycerophosphate, and 1 mM phenylmethanesulfonyl fluoride. In a separate experiment, mouse aortic tissues were isolated and used as an ex vivo model to examine the effect of ZEN on vessel relaxation. Dissected aortas were exposed to either 20 μM of ZEN or vehicle and incubated at 37 °C under 5% CO_2_ for 16 h. The aortas were then chopped in pieces with iris scissors and incubated in lysis buffer to extract aortic protein. The concentrations of aortic protein were assessed using a BCA assay. Equal amounts of isolated protein (20 µg) were separated using sodium dodecyl sulfate polyacrylamide gel electrophoresis (SDS-PAGE) and then transferred onto nitrocellulose membranes. Blots were tagged with the appropriate primary antibodies and the corresponding secondary antibodies. The blots were developed using enhanced chemiluminescence reagent (ECL, GE Healthcare, Pittsburgh, PA, USA).

### 5.9. Assessment of Nitric Oxide (NO) Production

NO production by BAECs was assessed by measuring the concentration of nitrite, a stable metabolite of NO, in cell culture supernatant as previously described [12]. The culture medium was removed, and the cells were incubated in Kreb’s buffer (pH 7.4; 118 mM NaCl, 4.6 mM KCl, 27.2 mM NaHCO_3_, 1.2 mM KH_2_PO_4_, 2.5 mM CaCl_2_, 1.2 mM MgSO_4_, 11.1 mM glucose) at 37 °C for 1 h. Each 200 µL of supernatant was transferred into a 96-well plate, and additional 100 µL of Griess reagent (50 µL of 0.1% *N*-(1-naphthyl) ethylenediamine and 50 µl of 1% sulfanilamide containing 5% phosphoric acid) was added into each well. The plate was incubated at room temperature for 15 min, and optical density was measured using a microplate reader at a wavelength of 530 nm.

### 5.10. In Silico Analysis

The putative binding sites of transcription factors in human eNOS promoter were predicted using an Alibaba 2.1 online tool (http://generegulation.com/pub/programs/alibaba2/index.html).

### 5.11. Chromatin Immunoprecipitation (ChIP) Assay

A ChIP assay was done using a ChIP assay kit (Merck Millipore), according to the manufacturer’s protocols as previously described [52]. Formaldehyde (1%) was used to cross-link the cells, and the reaction was quenched by adding 0.1 M glycine. The cells were then suspended in SDS lysis buffer (1% SDS, 10 mM EDTA, and 50 mM Tris-HCl at pH 8.1). The samples in SDS-lysis buffer were sonicated and precleared by protein A-agarose/Salmon Sperm DNA beads and then immunoprecipitated with 400–500 μg of sample and 2 μg of Sp1 or non-immune mouse IgG antibody as a negative control (Santa Cruz). DNA was isolated from total chromatin extract (Input) and ChIP samples and then used for PCR analysis. PCRs were performed using bovine eNOS promoter-specific primers: *eNOS*-F, 5′- CTC AGA GCG GAA CCC AGG -3′ and *eNOS*-R, 5′- AGC AGA GCT TCG GGC CTT -3′.

### 5.12. Preparation of Nuclear and Non-Nuclear Fractions

Nuclear and non-nuclear fractionations were carried out as previously described [53]. Cells were lysed in nuclear extract buffer I (NEBI) (10 mM HEPES at pH 7.9, 10 mM KCl, 1 mM DTT, 0.1 mM EDTA, 0.1 mM EGTA) containing protease inhibitor. The suspension was incubated on ice for 15 min and lysed with additional 0.5% NP-40 followed by vortexing for 10 s. The cell lysate was subsequently centrifuged at 16,000× *g* for 30 s, and the supernatant was collected and designated as the “non-nuclear fraction,” which was kept at 4 °C until use. The remaining pellet was re-suspended in NEBII (20 mM HEPES at pH 7.9, 400 mM NaCl, 1 mM DTT, 1 mM EDTA, 1 mM EGTA) containing protease inhibitor. The re-suspected pellets were incubated on ice for 30 min and centrifuged at 16,000× *g* at 4 °C for 20 min. The supernatant was collected and designated as “nuclear fraction”.

### 5.13. Co-Immunoprecipitation (co-IP)

The nuclear fraction (200 μg) of cells treated with ZEN or vehicle was immunoprecipitated overnight using 1 μg of specific antibodies against Sp1, PXR, or non-immune mouse IgG as a negative control (Santa Cruz) at 4 °C. Protein-G sepharose beads were incorporated into the samples and incubated for 2 h at 4 °C. After the incubation, the beads were washed three times with NEBII. Lastly, these samples were mixed with SDS loading buffer, boiled for 5 min, and prepared for Western blot analyses.

### 5.14. Animals

All animal experiments were carried out under the approval of institutional guidelines for animal care and use in Yeungnam University (YUMC-AEC2019-003). Male C57BL/6 mice (eight weeks of age) were housed in a temperature- and humidity-controlled cage (22 ± 1 °C and 50 ±10%, respectively) under 12 h alternating light/dark cycle for 1 week at the beginning of the experiment. All mice were fed standard chow (Purina Mills, St. Louis, MO, USA) ad libitum and water during the experimental trials.

### 5.15. Measurement of Endothelium-Dependent Vessel Relaxation

Endothelium-dependent vascular relaxation was determined in thoracic aortic rings as previously described [15], with slight modifications. Male C57BL/6 mice were sacrificed, and adventitial connective and fat tissues were carefully removed. The thoracic aorta was cut into 2 mm rings under a dissecting microscope and treated with either 20 μM of ZEN or DMSO as a control for 16 h. After the treatment, these rings were suspended by a small vessel wire myograph containing 37 °C Krebs-bicarbonate buffer (118 mM NaCl, 4.6 mM KCl, 27.2 mM NaHCO_3_, 1.2 mM MgSO_4_, 1.2 mM KH_2_PO_4_, 2.5 mM CaCl_2_, 11.1 mM glucose) in a chamber maintaining 5% CO_2_ at 37 °C. Isometric tension was measured by using a force transducer. Aortic rings were equilibrated in Krebs-bicarbonate buffer for 30 min under resting tension of 0.5 g and then constricted by adding 0.1 μM phenylephrine (PE) until a steady-state was reached. Endothelium-dependent relaxations were determined by measuring the dilatory response of arteries to acetylcholine (ACh) (from 0.001 to 1 μM). The endothelium of the aortic ring was considered to be intact when complete relaxation (100%) was observed with 1 μM ACh treatment.

### 5.16. Statistical Analysis

All of the results, except the ex vivo aorta relaxation assay, were expressed as mean ± standard deviation (S.D.) with *n* indicating the number of repeated trials. Results from the ex vivo aorta relaxation trial were expressed as the mean ± standard error (S.E.), with *n* indicating the number of trials. Statistical significance was determined using Student’s *t*-test for paired data. A value of *p* < 0.05 was considered significant.

### 5.17. Ethical Approval

In brief, the animal study we applied in the manuscript was approved by the Institutional Animal Care and Use Committee (IACUC) as follows. Approval number: YUMC-AEC2019-003. Title of Project: Effect of zearalenone and telmisartan on eNOS and muscle tension of arteries in vascular endothelial cells and smooth muscle. Periods of Projects: 2019. 4.15–2019.10.31 Date of Approval: 15 April 2019.
